# Comparing effects of tobacco use prevention modalities: need for complex system models

**DOI:** 10.1186/1617-9625-11-2

**Published:** 2013-01-22

**Authors:** Steve Sussman, David Levy, Kristen Hassmiller Lich, Crystal W Cené, Mimi M Kim, Louise A Rohrbach, Frank J Chaloupka

**Affiliations:** 1 Departments of Preventive Medicine and Psychology, University of Southern California, Soto Street Building 302A, 2001 N. Soto Street, Los Angeles, CA, 90033-9045, USA; 2 Department of Oncology, Georgetown University, Washington, DC, WA, USA; 3 University of North Carolina at Chapel Hill, Gillings School of Global Public Health, Chapel Hill, NC, USA; 4 University of North Carolina, School of Medicine, Chapel Hill, NC, USA; 5 University of North Carolina, Cecil G. Sheps Center for Health Services Research and the NCTRaCS Institute, Chapel Hill, NC, USA; 6 Institute for Health Research and Policy, Health Policy Center, University of Illinois, Chicago, IL, USA

**Keywords:** Relative effects, Tobacco use, Prevention

## Abstract

Many modalities of tobacco use prevention programming have been implemented including various policy regulations (tax increases, warning labels, limits on access, smoke-free policies, and restrictions on marketing), mass media programming, school-based classroom education, family involvement, and involvement of community agents (i.e., medical, social, political). The present manuscript provides a glance at these modalities to compare relative and combined impact of them on youth tobacco use. In a majority of trials, community-wide programming, which includes multiple modalities, has not been found to achieve impacts greater than single modality programming. Possibly, the most effective means of prevention involves a careful selection of program type combinations. Also, it is likely that a mechanism for coordinating maximally across program types (e.g., staging of programming) is needed to encourage a synergistic impact. Studying tobacco use prevention as a complex system is considered as a means to maximize effects from combinations of prevention types. Future studies will need to more systematically consider the role of combined programming.

## 

Tobacco use prevention efforts have a history extending back to advocacy and education at schools exerted prior to the first Surgeon General’s Report in 1964 [1,2]. Researchers entered the arena of tobacco use prevention primarily after release of that first report [3]. Research has been extensive since that time [2,4-7]. There are many avenues of prevention that have been implemented and evaluated. Tobacco use prevention efforts have been primarily focused on youths who are 11 to 18 years of age, with some exceptions (e.g., Jackson & Dickinson [8] with parents who are smokers and their eight year olds), and have been implemented at home (e.g., family, mass media), school (classroom-based or after-school), and other community settings (e.g., stores, clubs), through educational and policy efforts.

General statements about the efficacy of different types of programming have been made at several time points since release of the first Surgeon General’s Report (e.g., the first SGR on the prevention of tobacco use among young people [7], CDC Guide to Community Preventive Services [9], summarized in Task Force on Community Preventive Services [10]). Of recent importance, several notable consensus statements have been made since 2006. The NIH State-of-the-Science Conference statement [11] argued for three effective general population approaches to preventing tobacco use in adolescents and young adults: (1) increased prices through higher taxes on tobacco products; (2) laws and regulations that prevent young people from gaining access to tobacco products, reduce their exposure to tobacco smoke, and restrict tobacco industry marketing; and (3) mass media campaigns. This statement also concluded that school-based programs aimed at preventing tobacco use in adolescents are effective in the short term, and mentioned that comprehensive statewide tobacco control programs have also reduced overall tobacco use in young adults.

The Institute of Medicine (IOM)’s 2007 report [12] stated that the most fully developed programs for preventing youth tobacco use have been implemented in school settings, and that school-based programs should remain a mainstay of tobacco use prevention activities. This report also suggested that investing in programs for families and health care providers is warranted, even though the evidence base remains thin. Finally, the report recommended funding of mass media campaigns.

Next, the National Prevention Council’s report [13] was published in 2011. This report stated that effective strategies include adopting and enforcing comprehensive smoke free laws in multiple settings; implementing mass-media and counter-marketing campaigns; making options that help people quit accessible and affordable; and implementing evidence-based strategies to reduce tobacco use by children and youth. In addition, this report supported full implementation of the 2009 Family Smoking Prevention and Tobacco Control Act [14]. This act grants the U.S. Food and Drug Administration (FDA) authority to regulate the manufacture, marketing, and distribution of tobacco products. Falling under FDA authority, in principle, means that all nicotine-containing products, including cigarettes, are considered drug delivery devices subject to the same rigorous safety standards as other drugs.

Finally, the 2012 Surgeon General’s Report [2] concluded that that mass media campaigns, comprehensive (multiple type) community programs, and comprehensive statewide tobacco control programs can prevent the initiation of tobacco use and reduce its prevalence among youth. This report also concluded that increases in cigarette prices reduce the initiation, prevalence, and intensity of smoking among youth and young adults. Third, this report concluded that school-based programs with evidence of effectiveness, containing specific components, can produce at least short-term effects and reduce the prevalence of tobacco use among school-aged youth.

## The approach of the present study

We revisit different modalities of tobacco use prevention programming. “Modalities” of tobacco use prevention programming have been previously referred to as “types” of programming, program “components”, types of “policies”, “approaches”, or “strategies” [2,4,5]. Basically, “modalities” intends to refer to the different ways and settings employed to attempt to decrease incidence and prevalence of tobacco use among youth (e.g., policy-based, school-based, mass media-based). We use “types” and “modalities” of programming interchangeably in this paper. We attempt to speculate on the unique effect of each program modality. That is, for each modality, we estimate the difference in the percentage change in tobacco use as the result of exposure to the program/policy condition modality minus the percentage change in tobacco use under a control condition. We intend these effects to occur over at least a one-year post-program/policy period, assuming ideal delivery/enforcement. Of course, in some of the comparisons provided, effects were examined over as long as a five-year period (in the school-based work [2]).

## Methods

The studies were selected through three sources, mainly as a review of previous reviews. First, we included all Cochrane Reviews resulting from a search including the keywords “tobacco use prevention” or “smoking prevention.” Second, we included all major U.S. reviews on tobacco use prevention since 2000 that presented data on two or more modalities. Finally, we included previous reviews that the authors had been involved in since 2000 that presented data on two or more modalities. This search process led to a thorough set of published reviews that included most of the relevant studies.

We reconsider literature that reports outcomes utilizing similar analytic approaches, and attempt to consider a wide spectrum of program modalities. We do not consider unique populations (e.g., indigenous youth; [15]) or unique types of prevention programming (e.g., use of incentives; [16]), for which only a couple of research studies exist. Rather, we consider five general types of regulatory approaches/policies (tax increases, warning labels, access laws, smoke-free policies, and marketing/advertisement bans), and mass media, school, family, and community agent modalities of programming (see Table 1). In addition, we consider the results of community-wide approaches, which combine various modalities [2,17,18]. Finally, we offer structure to the search for synergic program combinations. We suggest directions that future research may take, including consideration of tobacco use prevention as a complex system. 

**Table 1:** Modality by incremental impact summary

	**Percentage Impact of modality**	**Implementation considerations**
Modality		
Tax-induced Price Increases	6% to15%	If 20% increase in price
Warning Labels	2%	Large (e.g., 50% of pack)
Access Restriction	2%	Enforced in retail contexts
Totally Ban Public Use	6%	Total
Totally Ban Ads	4% to 6%	Total
School	5% to 10%	With fidelity
Mass Media	4% to 7%	With other programming
Family	5% to 10%	If cooperate
Other Agents	2%	If actively involved

## Results and discussion

### Tax increases

A major type of policy used to prevent (and control) tobacco use is through tax increases that result in price increases. Tax or other price increases on tobacco products can curb youths’ intention to begin smoking, particularly if large increases are incurred. It has been estimated that a 20% increase in price leads to an incremental reduction of 6% to15% in smoking prevalence among youth [4,19-21]. For example, subsequent to a cigarette price jump of 22% after passage of the 2009 U.S. Federal Tobacco Tax, youth smoking (8^th^, 10^th^, and 12^th^ graders) decreased 14%. When examining price impact it is difficult to control for simultaneous programming and a large scale environmental demand reduction trend due to increased unpopularity of smoking. However, a sharp decrease in prevalence is suggestive of a dramatic event, plausibly the price increase [19].

### Use of warning labels

A second type of policy is use of warning labels. Warning labels placed on tobacco products using clear language in ways that cue behavior may provide a preventive impact. That is, youth may see the package warning language and visual imagery, and then feel frightened regarding potential consequences or at least be cued that tobacco use is dangerous, and then not smoke [2,22]. In particular, large graphic warning labels increase awareness of health risks of tobacco use, compared to text warning messages [2,22]. In the Framework Convention on Tobacco Control, 30-50% of the face of the cigarette pack is proposed as the desired size of graphic warning labels depicting disease consequences of tobacco use [23]. Large warnings labels may exert a 2% reduction in smoking prevalence [4]. A limitation of this type of programming would exist if youth are not notably emotionally impacted, or become habituated to labels and, hence, the effect would be weak or temporary [24]. Still, a 2% reduction may contribute to a multi-pronged impact on tobacco use.

Recently, the Food and Drug Administration (FDA) has attempted to mandate use of graphic large warning labels on cigarette packs in the United States, as has been done in several other countries [24], meeting a great deal of legal resistance from the tobacco industry [25]. Free speech, U.S. business law, and evidence for preventive efficacy of use of warning labels have been topics of debate [25]. Opposing appeal court decisions likely will be resolved at the level of the U.S. Supreme Court (Case #11-5332).

### Access-related tobacco prevention policy

A third policy action pertains to blocking access to tobacco sources among youth. This policy mainly addresses enforcement of the 1992 Federal Synar Amendment (Alcohol, Drug Abuse, and Mental Health Administration Reorganization Act [P.L. 102–321], amendment [section 1926]) which requires all States in the U.S. to prohibit the sale or distribution of tobacco products to persons under 18 years of age, and permits annual, unannounced inspections of tobacco sales outlets to discern noncompliance. If youths are denied access to tobacco at retail sources, they may be able to use tobacco less often. Indeed, the Synar program has resulted in decreases in self-reports of obtaining cigarettes through store purchases (40% in 1997 down to 9% in 2010, see [2] p. 711). Standard enforcement of youth access laws may lead to a 2% reduction in youth smoking prevalence [21], though some higher estimates have been made based on perfect compliance to no-access regulations (e.g., 25% reduction [4]). Youth are still able to locate non-compliant retailers that will sell to them or obtain tobacco through non-retail sources such as buying or stealing cigarettes from older siblings, other adults, and requesting older strangers to purchase tobacco for them [26], with standard enforcement.

### Smoke-free environment policy

A fourth type of policy modality is the enforcement of smoke-free environments where youth congregate. A no-smoking public environment law may lead to a reduction of up to 11% among adults, but perhaps only up to 4% to 6% for youth in monthly smoking (perhaps up to a 13% impact on weekly smoking [2,4,5,27,28]), in part because of inadequate enforcement, but also because youth are less likely to smoke in the public purview. In fact, adolescents are most likely to smoke cigarettes and use other drugs in their bedrooms at home [29].

Where antismoking norms have not been established or where there is not strong support, voluntary compliance may be less and enforcement may be minimal. In the absence of voluntary compliance, continued enforcement with meaningful fines may be needed. Certainly, media campaigns and other education oriented programs or policies may be needed to inform the public of the dangers of second hand smoke. These educational approaches may help increase voluntary compliance [30].

### Bans on tobacco advertisement

Tobacco use advertisement/marketing bans are a fifth type of policy action which may also dissuade youth from uptake of tobacco use [2,4]. Less public information aimed at persuading people to use tobacco helps decrease informational social influences conducive to use [31]. A full ban on ads may lead to a 4 to 6% reduction in smoking prevalence [4]. However, partial bans provide a much weaker impact, if any, and product placement (e.g., depiction of smoking in movies and other media) is an example of yet other channels of informational social influence to use tobacco (e.g., covert marketing), which also need to be controlled [5].

### Mass media impact

The mass media modality includes a variety of dissemination channels to provide a preventive effect. These channels include not only televised campaigns and print format Public Service Announcements (e.g., a new National media campaign was launched March 15, 2012 [32]), but also could include use of computers [33], the Internet, interactive CDs (e.g., video games), cell phones (e.g., text messaging), and SMART phones. Mass media-based prevention efforts are likely to be most successful when they involve novel, fast, unconventional portrayals (e.g., of health effects, passive smoking exposure, tobacco industry deception) to elicit social learning ([2,34] on pps. 194–198). Worden, Flynn, and colleagues conducted some of the more rigorous assessments of use of the mass media in studies of cigarette smoking prevention. Through creation of a matched pairs design of school versus school plus media conditions in two metropolitan areas in Vermont and New York, these researchers found that the media component provided a 2% to 8% incremental effect after four years of programming (2.6% versus 4.4% at least one cigarette per week smoking prevalence of school + media versus school only), and two years after that, when participants were in 10^th^ to 12^th^ grades (about 17% versus 25%, respectively). The findings from this study also were moderated by risk. Those at higher risk for continued smoking (they or their family member smoked at baseline) were impacted more strongly by the program (showed a relatively greater decrease in prevalence) than those at lower risk [35].

Snyder and colleagues [36] conducted a meta-analysis on the effect of mediated health communication campaigns on behavior change, all of which had involved use of at least one form of community-wide mass media. Youth tobacco use prevention campaigns showed a (small) effect size of .05 to .06 or about a 3% reduction in smoking if exposed to the campaign (also see [37]). The most up-to-date review [2], which also considers the impact of the TRUTH campaign [38], asserts a causal effect of the use of mass media campaigns, and a dose–response effect, of aggressively delivered campaigns. There are difficulties with assessment of mass media tobacco use prevention impact, including providing adequate control group comparisons, or examination of mass media impact in isolation from other types of programming. Given these limitations, we estimate that mass media programming may elicit a 4% to 7% incremental preventive effect [2,4,12,33,37-41] contingent on the adequacy of the reach of such programming, the opportunity provided for interaction about the media programming, and supplementation with other types of programming.

### School-based programming

School-based tobacco use prevention research has experienced a radically varying history in terms of perceived efficacy among the research community [6]. During the last 25 years of the twentieth century it was considered to be the most efficacious type of programming. Then, beginning approximately in the Year 2000, it was thought to not work except for a few exceptions [6]. Recently, beginning in 2008, it has received renewed interest and is recognized again as an important type of programming (e.g., see [6,42-46]). Schools have been a central means of delivery of programming because youth are a captive audience; though institutionalization at schools has not been solidly accomplished. This type of programming was the most widely studied in tobacco use prevention research up until around 2000 [6,31]. A barrage of publicity began a discrediting of school-based tobacco use prevention programming based on the evaluation of the Hutchinson Smoking Prevention Project (HSPP [47]) which was implemented in the state of Washington among a large sample of small, majority white, rural schools. The HSPP was a clustered-randomized controlled trial that was conducted from 1984 to 1999. The intervention tested was social influences-based, and was delivered to cohorts of youth from grades 3 to 12 in 40 randomly assigned school districts (n=8388). The analyses indicated that no significant overall differences were found in prevalence rates of smoking between participants in program and control districts in 12^th^ grade and 2 years after high school. A closer look at the data indicated that there were district effects, some positive and some negative. In addition, it was not clear if the program even produced short-term (e.g., 1-year post-implementation) effects, which would have dissipated. It remains unclear how to interpret the lack of overall effects from this study [2]. Subsequently, Skara and Sussman [45] conducted an empirical review of school-based tobacco and other drug use prevention and found that 15 of 25 programs, at an average of 5-years follow-up, produced at least one positive effect on tobacco use comparing program to control groups. An analysis of program-control percentage differences in initiation of smoking over time revealed an average 11% incremental effect.

The most recent review of school-based approaches [2] suggests that school-based programs which use interactive delivery methods and take a broad comprehensive social influences/life skills approach with relatively more sessions can demonstrate a one-or-more year incremental reduction of approximately a 5% to 7%. Based on that report and the Skara & Sussman [45] review, we suggest a 5% to 10% incremental effect (program minus control condition difference) over an average 5-year post-program period.

### Family involvement

Family-involvement tobacco use prevention programming that includes more than five sessions or contacts, and instructs one on how to be a good role model, parental monitoring, contingency management, and parent–child communication skills can impact lifetime or more frequent smoking in families that are willing to participate (which may not be easy [48]), particularly with at-risk youth. In a recent meta-analysis of 20 randomized controlled trials (RCTs) of family-based smoking prevention, considering only those nine trials at minimal or moderate risk of bias and with at least a six-month follow-up, Thomas, Baker, and Lorenzetti [49] found that only four studies that tested a family intervention against a control group demonstrated significant positive effects. One of the five RCTs that tested a family intervention against a school intervention detected significant positive effects. None of the six RCTs that compared the incremental effect of a family + school intervention to school intervention-only, nor one RCT that compared a family tobacco to a family non-tobacco intervention, detected significant effects. We suggest that under optimal conditions, one might find a 5% to 10% incremental effect of family-based programming on youth tobacco use onset or increases.

### Community agents

Provision of prevention messages or endorsement of specific tobacco use prevention programs by medical, social, or political agents or leaders (“champions”) is another means of trying to prevent tobacco use. Community agents may include dentists, pediatricians, youth club leaders, local health service personnel, or city leaders (e.g., Michael R. Bloomberg [50]). While their time to assist in this endeavor is limited, and the effects they exert consequently are likely to be small, community agents come into contact with many families and are often respected by youth. In general, the few studies conducted in this arena have examined providing brief advice on different topics (e.g., tobacco use and sports, nicotine addiction), and providing direct messages not to start smoking or to quit, sometimes involving parents. Thus far most trials involving community agents have been disappointing [2], and we conjecture that at most a 2% incremental effect might be found (with a few exceptions). Combined with other types of programming, however, involvement of community leaders may be very important [17,51]. Arguably, this type of programming might be best considered as an aspect of another type of programming (e.g., as a “voice” in a mass media campaign). However, use of community leaders in tobacco use prevention has been considered a separable aspect of programming [2,51], and we feel that its delineation is conceptually useful (e.g., there may be different types of programming that include or do not include use of community agents). On the other hand, in a vast majority of instances community champions were not introduced as part of controlled trials, or as a separable feature within the program condition of a controlled trial. It would be possible to compare community champions or systematically include or not include such persons in trials. But this is unlikely to be accomplished in future work. Rather, we can only conjecture regarding their importance and attempt statistical means to try to gauge their impact.

### Tobacco industry youth prevention programming

One additional community agent to consider is representation of the tobacco manufacturing industry. The tobacco industry has continued to respond with various types of youth tobacco use prevention methods [52]. For example, Altria’s current tobacco use prevention programming includes under-age access restriction and age verification (We Card), mandated disengagement from “paid” product placement, due to the Master Settlement Agreement [53]; deletion of cartoon billboard or stadium ads; deletion of brand name sponsored concerts, merchandise with brand names in the U.S., or provision of samples of cigarettes; very limited modes of impersonal sales (e.g., no more deployment of vending machines); and funding of positive youth development grants (provided to major youth organizations in U.S.) such as ParentFurther [54].

There still appears to be the suggestion by the tobacco industry of tobacco use as being a mature, responsible choice among adults, which presents the possibility of a forbidden fruit motivation [55]. Adolescents who perceive cigarette smoking (or other tobacco use) to be socially approved adult behavior (a “responsible” choice) may over time develop intention to smoke cigarettes, as they grow older, and question whether or not the concept of forbidden fruit (i.e., only for adults, not children) should apply to them, as was suggested in a recent study [55].There is a paucity of evidence that the tobacco industry contributes much *voluntarily* to the community agent effect, and generally the industry is seen to undermine tobacco control efforts [2].

### Multiple-modality community-wide programming

In principle, “flooding the field” with tobacco use prevention programming and policies help maximize preventive efficacy [2,17,18,51,56]. Flay [6] suggested that school plus community programs could double the effect of school-only programs. However, his paper only included discussion of five community-level studies. Unfortunately, several community-based efforts have failed to find programmatic effects [2,17,57]. Of particular relevance, Carson et al. ([17]; also see the review preceding it, [58]) completed a recent systematic review of community-based programming involving multiple types. Twenty-five studies were included in the review (68 other studies did not meet the inclusion criteria). All included studies used a controlled trial design, with 15 using random allocation of schools or communities. Eleven of 25 studies reported tobacco prevention effects. These authors concluded that coordinated, multi-modality community programs may be able to reduce smoking among young people, depending on the combination of strategies used and penetration of the strategies [17]. Likely due to the heterogeneity in combined interventions across reviewed studies, the authors make a minimal attempt to specify the most successful combination or sequencing of interventions; they rather advocate the use of programs for which effectiveness has been demonstrated, that are acceptable to the community, and that are “guided by a combination of theoretical constructs about how behaviors are acquired and maintained” (p. 19 [17]).

An examination of the review by Carson et al. (Table two [17]) suggests an average effect advantage of multi-component community-wide programming versus a control condition of 4.7%, in part due to relatively large effects of four studies [51,59-61]. One suggestion from these findings is that effects of multi-modality community programming, in general (with a few notable exceptions), are no stronger than with implementation of single component programming. Possibly multi-modality community programming may permit better maintenance of program effects, though even this point is not definite [45]. The programs that did exert the strongest results tended (a) to involve community organizers or champions closely aligned with the project as supporters, (b) addressed cardiovascular risk reduction as well as tobacco use prevention or otherwise targeted multiple behaviors, (c) provided means of tobacco use cessation as well as prevention, and were (d) extended over a relatively long period of time (possibly involving staging in implementation of different modalities).

Community-based programs include many different types, such as statewide or multi-state efforts, and within-state community-based efforts involving different sized communities, all that may involve a variety of different modalities of programming in the mix, with utilization of different types of research designs. Thus far, it has not been possible to discern the relative impacts of these variations in implementation design. Another point to consider is that price and enforcement of no-smoking policy measures generally were not assessed as part of these multi-component community-wide programs. It is possible that combining these regulatory policy-based measures with community-wide programming is a *sin qua non* to producing long-lasting large decreases in tobacco use prevalence over time [2].

### Considering incremental effects of tobacco use prevention program modalities

Certainly, there are many limitations when trying to examine effects across different types of tobacco use prevention modalities. First, we gauged effects based on previous reviews’ overall effects (some involving meta-analytic derived estimates or simulations), or through simple averaging of differences across studies which provide a rather gross estimate (e.g., we didn’t consider standard errors of estimates). The reviewed findings may be confounded with other factors, such as the social norms regarding smoking in different states or countries. Also, there is the possibility that modality incremental effect estimates may actually reflect synergic or dampened effects due to trying to isolate the impact of the modality in the context of other social events or modalities.

Second, it is difficult to measure the impact of several of these types of programming in general. For example, price increases should provide a preventive impact on adolescents. However, there are relatively few national level datasets to provide appropriate data for archival-type analysis among teens. As another example, comparison conditions are difficult to create for mass media programming. As yet another example, school-based research study impact is unlikely to reflect impact under more usual conditions of delivery (dissemination-related impact). Also, family-based tobacco use prevention programming may provide a 5% to 10% impact on tobacco use under ideal conditions. However, it is difficult to enroll a majority of families in such programming or evaluate it.

Third, it is difficult to compare modalities, considering that estimates are derived of necessity from different types of research designs. For example, one can do a randomized trial of a school-based intervention, but not of a tax increase or a smoke-free policy. In the former case, one may examine changes in program and control groups directly. In the latter case, one suggests that tobacco use prevalence change would reflect naturally occurring increases in tobacco use over time if no policy was added. This may not be true since many simultaneous events may impact on tobacco use prevalence. However, the idea still is to compare changes in a program condition (some percentage decrease in tobacco use due to a policy or program) minus changes in a control condition (e.g., a 3% increase in last 30-day smoking per year [31]). This approach, or approaches like this one, have been considered previously through use of simulation modeling, meta-analysis, or empirical review of rough mean estimates [2,4,5].

A final issue regarding the difficulty in measuring the impact of modalities pertains to their comparative cost-effectiveness. The cost-effectiveness of different modalities is of importance, and will surely figure into policy makers’ decisions on which modality or combinations of modalities to implement. Also, there have been some cost-effectiveness studies on some modalities or specific programs within modalities (e.g., youth access ([62]; $44 to $8,200 cost per year of life saved), smoking restrictions ([63]; $42-$78 billion total savings in U.S. due to a national smoke-free environment), mass media programming ([64]; adjusted program cost per smoker averted $6069), school-based programming ([65]; savings of $13,316 per year of life saved), and taxation ([66]; $20 average cost for death and disability-adjusted life years averted). Certainly, some interventions are relatively low cost and highly effective, so relatively cost-effective (e.g., besides costs of enactment: taxation, smoke-free public areas, and graphic warning labels). Others are higher cost but also cost-effective relative to yet other health interventions (mass media public education campaigns, school-based prevention, and youth access restrictions). However, there is dearth of studies in the cost-effectiveness literature on tobacco use prevention. Also, it is difficult to gauge the comparative cost-effectiveness of the modalities presented. Gauging the costs of enacting a law to guide imparting a modality, publicizing and implementing or enforcing a modality, evaluating the modality within and over time, and assessing the shelf-life of a modality (e.g., a mass media program can “get old”) is complex; and calculation and type of cost-effectiveness statistics used vary. Thus, a great deal of error variance exists in making estimates of type of programming effects. Finally, the mere analytic complexity of examination of multiple-type effects simultaneously makes it difficult to discern maximal combinations of types of programming.

Evaluation challenges aside, the fact that community-wide multi-modality programming provided effects that in a majority of cases was not stronger than single-modality type programming, suggests that either (a) there were key modalities missing from many of these trials, (b) that there were countervailing forces in operation, programming was implemented at inappropriate youth developmental levels, or in an unsupportive larger social environment (e.g., see [67]), or (c) that somehow certain otherwise successful components might have operated negatively together within the system of application.

More generally, it is not clear how different modalities will combine to produce program effects. For example, it is possible that one type (e.g., mass media) may provide a maximum subjective impact on youth, such that adding a second component (e.g., school-based classroom education) would not provide an incremental effect (*ceiling effect*). As more modalities are included, it becomes more likely that a subset will produce a ceiling effect and any others will provide no additional impact. A second possibility is that of a *potentiating effect*, in which one modality may exert little impact except when combined with another modality. For example, a mere “push” by a community leader may exert no effect unless it is associated with a highly credible community-based program. Third, it is possible that two or more components might all contribute *additively*. Inclusion of more and more types of programming would result in increasingly strong effects. Fourth, the components may, in some cases, provide a *synergistic impact*. That is, it is possible that greater than additive (for example multiplicative) effects may result from monopolizing the social environment with multiple modalities. Finally, it is possible that one type of programming could detract from another; that is, the effect of one type of programming may negate the potential impact of the other (an *antagonistic effect*). For example, one might envision, at least in some cases, parents wanting to be the sole provider of prevention efforts with their children and resenting involvement of the school, which might lead to undermining the impact of the school component. Of course, it is possible that any number of these five types of effects (ceiling, potentiating, additive, synergic, or antagonistic) could occur when considering any number of modalities.

### Future directions of tobacco use prevention research

There is still considerable room for progress on implementation of individual program modalities. For example, taxes are well below the levels recommended by the World Bank/World Health Organization; many populations are not covered by comprehensive smoke-free policies (all worksites, bars, restaurants and other public places); smoke-free areas might be extended to other venues (e.g., cars with children present, outdoor spaces like parks and beaches, or multi-unit housing); also, public education campaigns and other efforts supported by state programs have experienced funding cuts and are way below what Centers for Disease Control and Prevention recommends [2,53,66]. There is much more that can be done to prevent the health consequences of tobacco use through maximizing the impact of single modalities [2].

Second, few efforts have attempted to apply programming to all types of tobacco products [68,69]. For example, a few recent studies have found an inverse relationship among adolescents between product-specific tobacco taxes (or prices) and the propensity to use smokeless tobacco, the intensity of its use, and the prevalence of cigar smoking [2]. With the new FDA authority (Family Smoking Prevention and Tobacco Control Act), the next immediate new frontier for tobacco control appears to be product modification, such as reduced nicotine cigarettes, cigarettes with less harmful constituents, or safer tobacco products [14]. However, these developments could backfire for tobacco use prevention efforts -- if youth would now feel that smoking uptake is less risky due to perceived increased ease of quitting, or reduced harm of alternative tobacco products. Another policy that is being considered is removing flavor additives and menthol, because these might make tobacco products appear more like candy to youth [70]. Elimination of mentholated cigarettes could have a major public health impact due to smoking prevention or cessation among future or current menthol cigarette smokers [71]. Application of various prevention modalities to all tobacco products may be needed to provide a uniform impact on multiple tobacco products.

Third, it is not clear how multiple avenues of programming may interact with each other. The five potential models of effects of two or more types of programming (ceiling, potentiating, additive, synergic, or antagonistic effects) may all operate given different combinations of various types of programming. New evaluation directions are needed to be able to discern which combinations of program modalities to use (and in what order) to obtain maximum impacts. Very few program mediation analysis studies have been reported, but might provide direction on how to combine modalities efficaciously. Among the few studies that have been completed, norms manipulation (of prevalence or acceptability) and outcome expectancies manipulation (such as personally relevant consequences) have been found to mediate program effects (e.g., [72,73]).

### Complex systems

Efforts to prevent tobacco use would benefit from conceptualizing tobacco use and its prevention as a complex system. Although there is no common definition, a complex system is typically thought of as an entity composed of many different parts that are interconnected in a way such that the behavior and characteristics of the system as a whole cannot be understood or anticipated from analyzing its components alone [74]. Many factors can contribute to this complexity including: interrelated components with bidirectional “feedback” loops, relationships among some components not being linear (for example, threshold or ceiling effects), impacts stemming from multiple levels of influence, or there being heterogeneous and often long time delays between cause and effect [75]. In complex systems, small changes (e.g., implementation of a few modalities) can result in large effects and large changes (e.g., implementation of many modalities) may result in small or no effects, and it can be hard to predict which is more likely. Tobacco use prevention is an example of a complex system; examples of each of these characteristics contributing to complexity abound [76,77]. For example, tobacco use prevention strategies must consider the dynamic interplay between factors at multiple levels [2] including: *individual* (e.g. genetics, personality characteristics); *micro-social* (e.g. parental role modeling, social network characteristics, social norms); and *macro-social* (e.g. school systems, advertising campaigns, agricultural initiatives, political parties, political action). Effectiveness of multiple interventions within and across levels has been demonstrated, targeting a multiplicity of mediating pathways. With this many options, identifying the best combinations of these interventions is daunting.

It is certainly more challenging to study combined intervention programs in complex systems, such as tobacco use prevention, that are “blessed” with many evidence-based options. Fortunately, methods exist to support the analysis of complex systems. A good starting point is the creation of system models based on experience and available data to unpack the “black box” of intervention effects, explaining how intervention modalities lead to effects alone and in various combinations. These models are essentially dynamic system-level hypotheses. They often begin as qualitative diagrams speculating how the system behaves, but many are then quantified – through parameterization of a system of equations or a computational model. Once developed, these “system hypotheses” can be tested against new data, with the goal of evaluating consistency between hypothesis and new real world experiences of implementing combined intervention programs. Though it is not possible to “prove” the validity of a model, the more consistent these hypotheses are with real world experiences, the more confident one becomes in them and the more useful they become in guiding future intervention decisions.

Much can be learned through testing and building confidence in complex system models. First, they offer formal notation for making one’s own mental model explicit regarding how complex systems behave and for offering related hypotheses for discussion. Inconsistent models challenge one to revise hypotheses to better match the complete body of evidence. Once constructed, additional simulation and analysis of computational models can be conducted to: (1) support learning about the relative importance of determinants of a complex system’s behavior (for example, see [78]); (2) evaluate the extent to which insights regarding system behavior or modality conclusions are robust to uncertainties in the model, allowing prioritization and valuation of further data collection efforts; and (3) provide virtual “trials” comparing the effects of interventions over time on outcomes of interest such as the prevalence of smoking or other tobacco use [75]. Simulated interventions might involve single or combination approaches, with varying levels of intervention dose or reach.

In tobacco control research there are many examples of effective use of computational modeling, though often developed to study interventions one-at-a-time. An example of a computational model that has been used to study some combined interventions is the SimSmoke tobacco control simulation model. This model projects smoking rates and deaths attributable to smoking (in total and for lung cancer, COPD, heart disease, and stroke), and examines the effect of tobacco control modalities on those outcomes. The model has been used to examine the effect of modalities individually and in combination as a function of varying demographics. The model has been used for predictive/planning purposes, justification of policies individually or as part of a comprehensive tobacco control program, and to help facilitate understanding of the role of tobacco control policies and how they may be most effectively implemented [79]. The SimSmoke model has been shown to predict smoking prevalence well for different states and nations, though relatively little work has been focused on tobacco use prevention *per se*. The model appears to predict best in states and nations with strong tobacco control policies (e.g., [80-82]).

Existing simulation models do capture some nuances involved when prevention modalities are combined. For example, it is quite feasible to simulate the mechanisms affecting synergy when one program modality is combined with a second -- that decreases the target population for the first. As a concrete instance, if both mass media campaigns and school-based programs reduce the number of youth likely to initiate smoking, each will reduce the number in the target population for the other. Conversely, it is feasible to simulate how a program modality might increase the target population for another. As a concrete instance, when a mass media campaign increases the number of current smokers interested in quitting, it may increase the target population for a second intervention making accessible pharmacotherapy to support smoking cessation. More complex and likely real interactions between program modalities warrant further study.

Though real world evaluation of combined programming is preferable to simulation, trials of community-based multi-modality programs are challenging for many reasons. These include the tremendous heterogeneity of community program contexts [17] and the enormous number of modality combinations that would have to be evaluated (which might make such an analysis prohibitively expensive to complete). Integrating theory and past experience through system models could help narrow the number of program modality combinations that would need to be evaluated. To better equip existing and new computational models to support such an analysis plan, one needs to do more to unpack the “black box” currently limiting understanding of mediation of modality and modality combination effects. One promising line of investigation involves the use of theory and systems diagrams to inform the search for synergy in intervention combinations, suggested by Weiner and colleagues [83].

**Figure 1:**
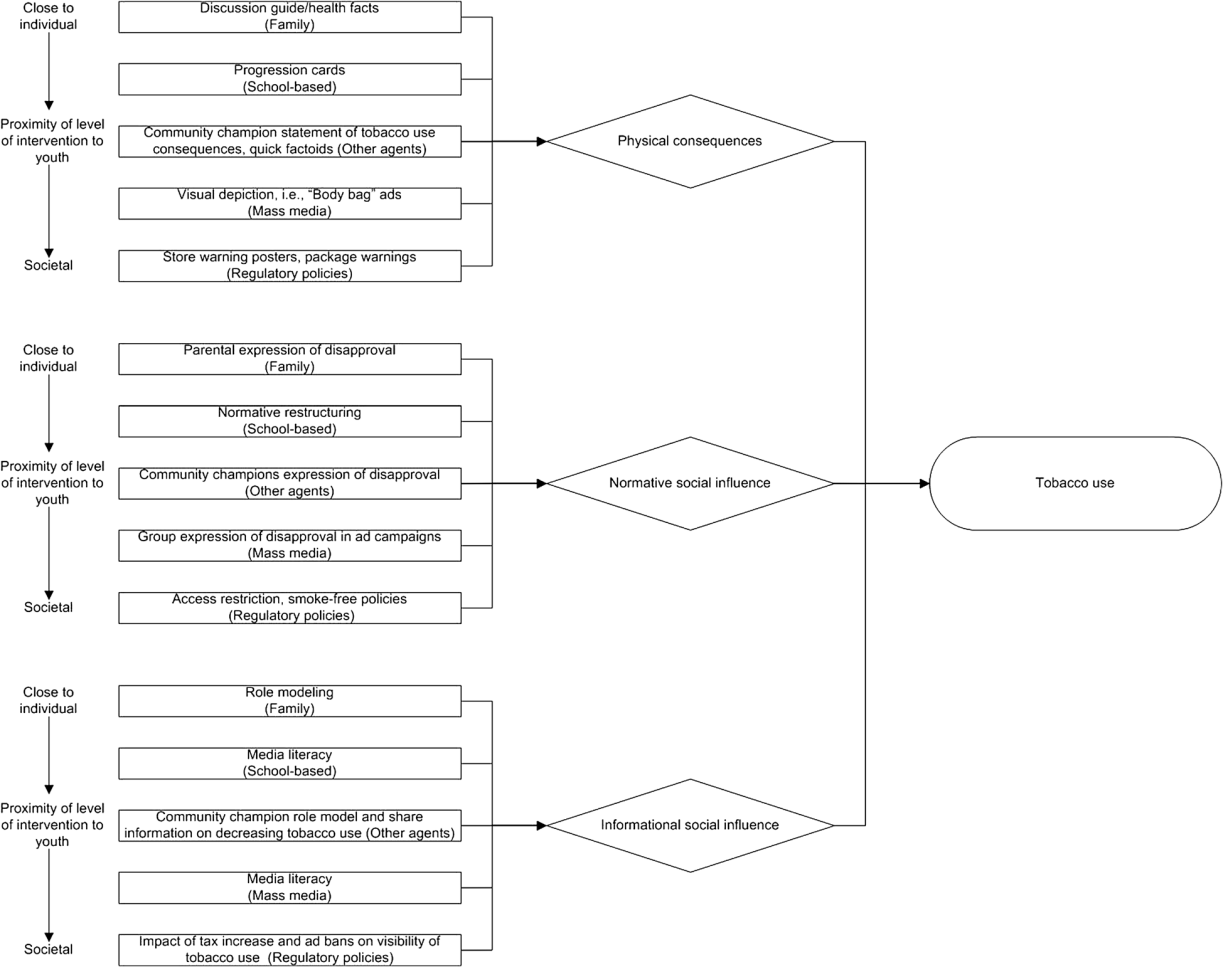
Illustrative accumulation strategy system diagram mapping program modalities (rectangles, with socio-ecological level in which modality is placed) to targeted mediating pathways (diamonds) to prevent or reduce tobacco use (oval).

**Figure 2:**
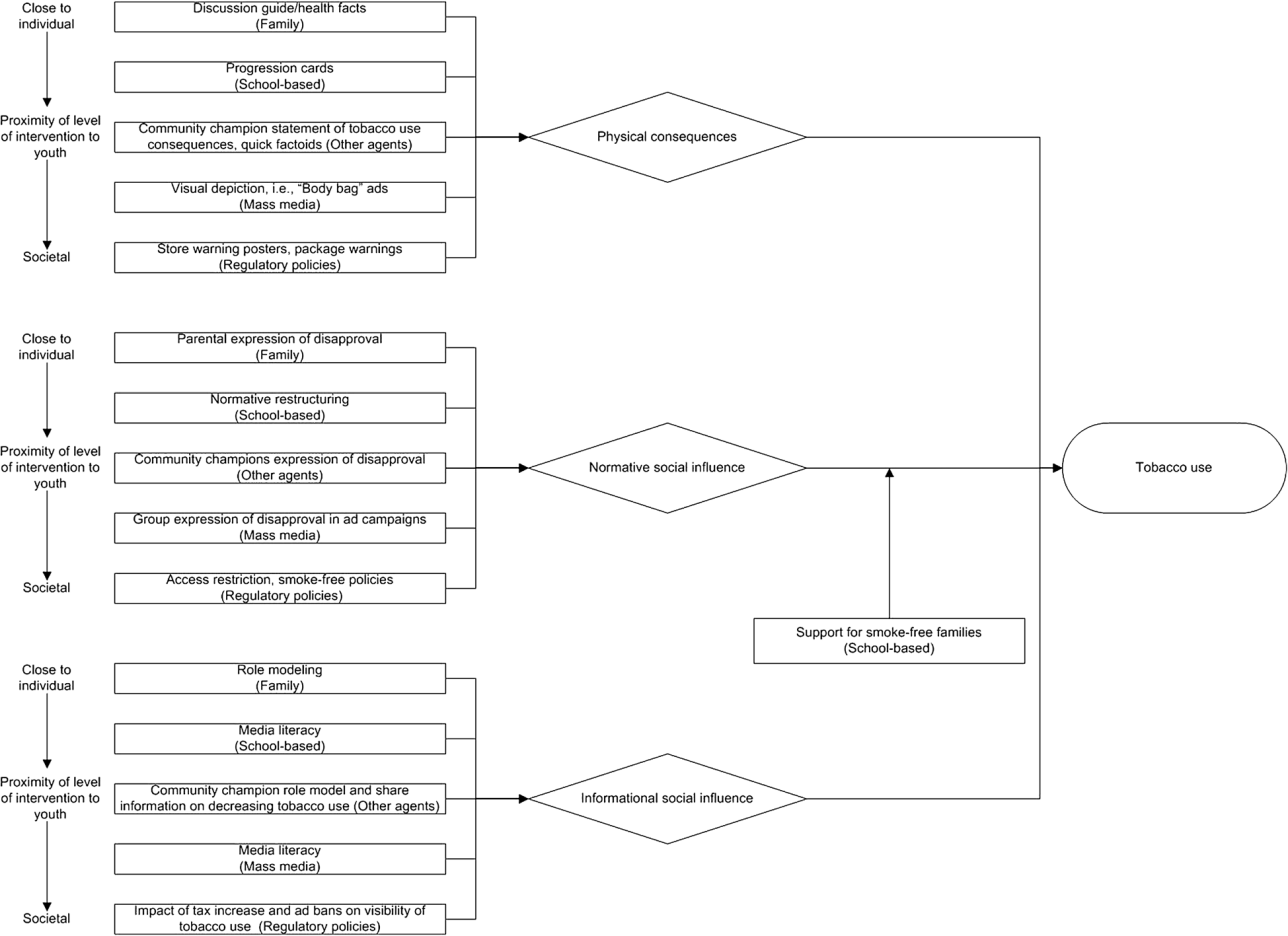
Illustrative facilitation strategy system diagram mapping program modalities (rectangles, with socio-ecological level in which modality is placed) to targeted mediating pathways (diamonds) to prevent or reduce tobacco use (oval).

**Figure 3:**
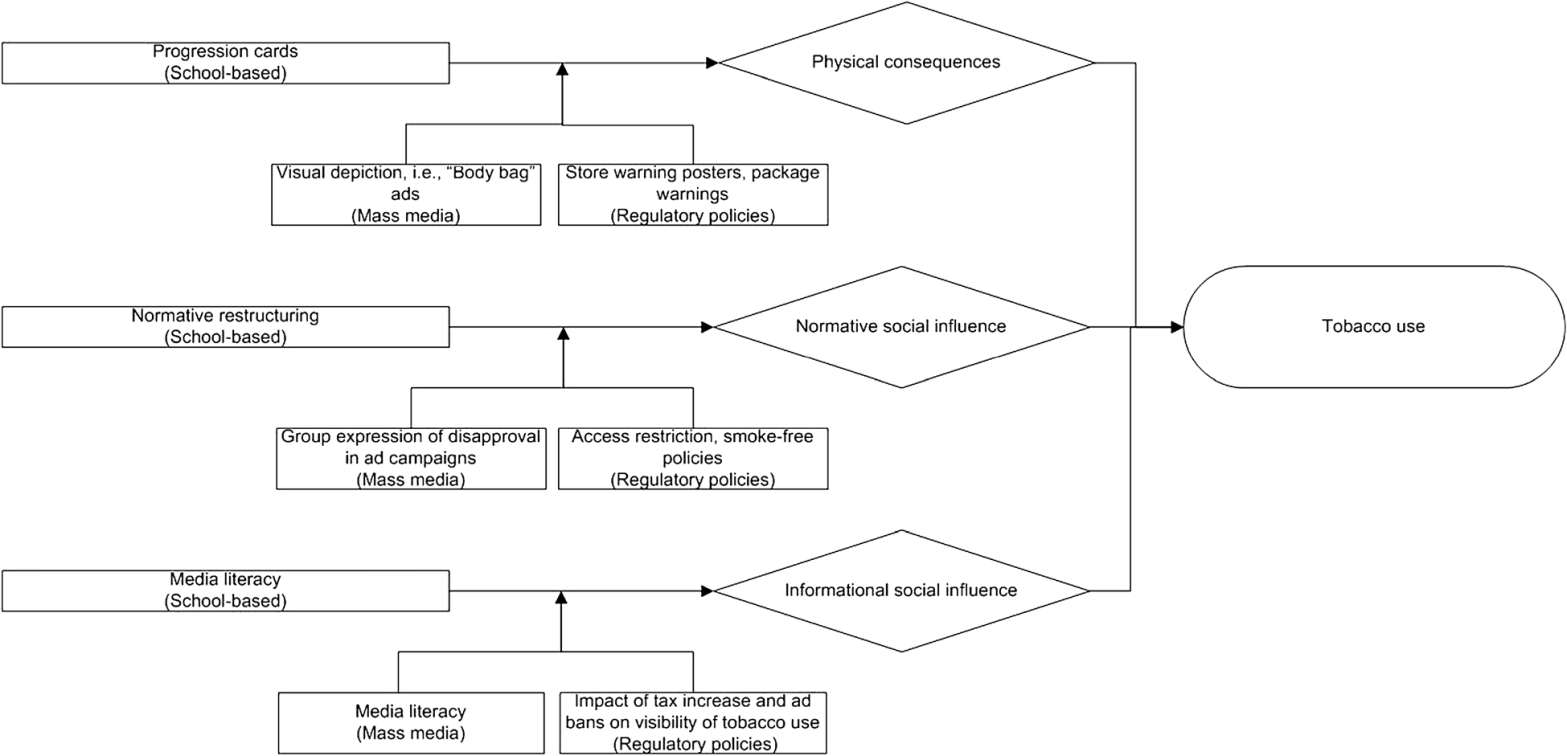
Illustrative amplification strategy system diagram mapping program modalities (rectangles, with socio-ecological level in which intervention is placed) to targeted mediating pathways (diamonds) to prevent or reduce tobacco use (oval).

Providing guidance to those seeking to combine interventions implemented across socio-ecological levels in cancer treatment, Weiner, Lewis, Clauser, and Stitzenberg identified mechanisms by which combining interventions is more likely to lead to synergy [83]. An illustration of what three of these strategies (i.e., accumulation, facilitation, and amplification) might look like in the context of tobacco use prevention is offered in Figures 1, 2, 3. All three figures map specific strategies from program modalities to mediating pathways, each, in turn, targeted to prevent or reduce tobacco use. The program modalities were family, school, community (other) agents, mass media, and regulatory-based. The strategies utilized were selected so as to map on to specific mediating pathways. The mediating pathways were derived from work completed by the lead author [31] on counteracting tobacco use by engaging in strategies serving to (a) increase perceptions of short-term and long-term physical harm resulting from use (physical consequences), (b) lower perceived or actual acceptability of use (normative social influence), and (c) lower perceived or actual estimates of frequency of use or discount social images associated with use (more covert, informational social influence). Thus, for example, “progression cards” refers to a specific school-based activity that depicts developing of addiction and disease through tobacco use, relevant to the physical consequences mediating pathway [31]. The specific regulatory mechanisms differed by specific hypothesized mediating pathway as well. For example, an advertising ban might decrease the perceived prevalence or certain social images (e.g., sex appeal, being older) associated with tobacco use, relevant to the informational social influences mediating pathway. These diagrams are speculative and incomplete, but meant to illustrate specific complex system strategies. Figure 1 represents the “accumulation strategy” for finding synergy in multi-intervention programs introduced by Weiner and colleagues [83] in which intervention synergy is sought through seeking multiple interventions that work through the same mediating pathway(s). Weiner encourages the selection and implementation of interventions to maximize the extent to which they “converge upon” the same target audience while avoiding ceiling effects. 

Figure 2 represents the “facilitation strategy” for finding synergy in multi-intervention programs in which intervention synergy is sought through adding an intervention capable of “clearing the mediating pathway” for the other interventions. In this speculative example, a program designed to prevent or reduce smoking in youths’ homes might make possible other prevention modality efforts to impact on the mediating pathways (i.e., demonstrate physical consequences, or counteract normative or informational social influences to use tobacco). For simplicity, in the diagram, an arrow is directed on one of the pathways (to normative social influence).

Finally, Figure 3 represents the “amplification strategy” for finding synergy in multi-intervention programs, through increasing the target audiences’ receptivity to the other interventions. In this example, macro-social factors, mass media campaigns and regulatory policy, would be implemented first to “prime” youth to amplify the effect of school-based program modalities that target each mediating pathway considered. In other words, these molar-level impacts could strengthen the impact of a more molecular modality (school-based programming) because they “prime the pump” for molecular-level activities that target the different mediating pathways. Further study and elicitation of such causal models could be built into computational models, which would then offer all of the benefits of computational modeling described above to informing multi-level intervention design.

Tremendous insights are likely to be gained from new models developed to better understand why integrated programming in certain contexts seem to be having less than expected and less than synergistic tobacco use prevention effects, and, more importantly, to inform the design of more effective multi-level, multi-modality tobacco use prevention programs. Figures 1, 2, 3 highlight the contrasts of tobacco use prevention program modalities described in this paper. However, many additional programmatic contrasts beyond those discussed here merit study, such as tobacco industry actions contrasted against tobacco use prevention policy enactment or enforcement (e.g., see 2, pps. 563–564;[76,84]). Future work might include an even more “molar” view to support more complicated decisions we now face about how to allocate limited resources across a variety of multi-level interventions to address tobacco use prevention, and also counteract tobacco industry attempts to undermine the effectiveness of different types of tobacco use prevention modalities. In future research studies, we expect that a better understanding of multi-pronged tobacco use prevention programming (contrasted with other social forces) will result as a function of utilization of complex systems as a framework, and that we will be able to maximize the interplay and impact of program types on youth tobacco initiation and increases.

## Conclusion

In a majority of trials, multi-pronged community-wide programming has not been found to achieve impacts greater than single modality programming. Certainly there are many variations in what constitutes a single modality of programming, and there are a myriad of different combination of programming modalities. Attempting to group programming within types may be difficult (e.g., mass media campaigns may be rather different from use of mass media television programs in specific settings), and grouping combinations of single-modality types of programming into a community-wide type of programming is difficult given the many parameters involved (e.g., size of community unit, types and dosages of programming included, relative single-modality cost-effectiveness). Still, considered across several consensus reports, empirical reviews, meta-analyses, and simulation studies, some idea on incremental efficacy is revealed. Importantly, for future research and practice, examination of tobacco use prevention as a complex system may be needed to maximize effects from combinations of modalities of prevention programming. Future studies will need to more systematically consider and uncover the combination rules and related incremental effects underlying efficacious multi-pronged community-based programming.

## Competing interests

The authors claim no competing financial or person interests with other people or organizations.

## Authors’ contributions

SS took the lead effort on this review paper, including the literature inclusion and writing. DL, FJC, and LAR provided critical read-throughs, suggested additional literature, and provided edits to improve the comprehensiveness and accuracy of the manuscript. DL and FJC were authors on an earlier review which attempted incremental impact estimates and provided expertise on policy modalities. LAR provided expertise on community-based research. KMH, CCW, and MMK also provided critical read-throughs and contributed most of the material on complex systems. All authors made substantive intellectual contributions to this paper. All authors read and approved the final manuscript.
